# The Genotypic and Phenotypic Characteristics Contributing to Flomoxef Sensitivity in Clinical Isolates of ESBL-Producing *E. coli* Strains from Urinary Tract Infections

**DOI:** 10.3390/antibiotics12030522

**Published:** 2023-03-06

**Authors:** Kazuma Sakaeda, Takuya Sadahira, Yuki Maruyama, Takehiro Iwata, Masami Watanabe, Koichiro Wada, Motoo Araki

**Affiliations:** 1 Department of Urology, Graduate School of Medicine, Dentistry and Pharmaceutical Sciences, Okayama University, 2-5-1, Shikata-cho, Kita-ku, Okayama 700-8558, Japan; 2 Koichiro Wada Department of Urology, School of Medicine, Shimane University, 89-1, Enya-cho, Izumo 693-8501, Japan

**Keywords:** antimicrobial resistance, *Escherichia coli*, urinary tract infections, flomoxef, ST131

## Abstract

We carried out a molecular biological analysis of extended-spectrum β-lactamase (ESBL)-producing *E. coli* strains and their sensitivity to flomoxef (FMOX). Sequence type (ST) analysis by multilocus sequence typing (MLST) and classification of ESBL genotypes by multiplex PCR were performed on ESBL-producing *E. coli* strains isolated from urine samples collected from patients treated at our institution between 2008 and 2018. These sequences were compared with results for antimicrobial drug susceptibility determined using a micro-liquid dilution method. We also analyzed cases treated with FMOX at our institution to examine its clinical efficacy. Of the 911 *E. coli* strains identified, 158 (17.3%) were ESBL-producing. Of these, 67.7% (107/158) were strain ST-131 in ST analysis. Nearly all (154/158; 97.5%) were CTX-M genotypes, with M-14 and M-27 predominating. The isolated strains were sensitive to FMOX in drug susceptibility tests. Among the patient samples, 33 cases received FMOX, and of these, 5 had ESBL-producing *E. coli*. Among these five cases, three received FMOX for surgical prophylaxis as urinary carriers of ESBL-producing *E. coli*, and postoperative infections were prevented in all three patients. The other two patients received FMOX treatment for urinary tract infections. FMOX treatment was successful for one, and the other was switched to carbapenem. Our results suggest that FMOX has efficacy for perioperative prophylactic administration in urologic surgery involving carriers of ESBL-producing bacteria and for therapeutic administration for urinary tract infections. Use of FMOX avoids over-reliance on carbapenems or β-lactamase inhibitors and thus is an effective antimicrobial countermeasure.

## 1. Introduction

*Escherichia coli* (*E. coli*) is the most frequently detected pathogen in urinary tract infections (UTIs), accounting for 50% to 85% of cases [1,2]. Furthermore, the frequency of bacteria producing extended-spectrum β-lactamase (ESBL) has recently been increasing worldwide, including in Japan, and antibiotic resistance is becoming a larger problem [3,4]. The abuse of broad-spectrum antibiotics is one of the major causes of the development of antimicrobial-resistant bacteria. The problem of antimicrobial resistance has become a public threat. The frequency of occurrence of UTIs caused by ESBL-producing *Enterobacterales* has been increasing globally. Among ESBL-producing *Enterobacterales*, ESBL-producing *E. coli* is considered the greatest concern [4].

ESBL-producing bacteria are drug-resistant organisms that carry genetic mutations in the enzyme that degrades β-lactams by hydrolyzing them into cephalosporins and monobactams. The ESBL gene is encoded on a transmissible plasmid and is widely transmitted horizontally to mycobacteria of different *Enterobacterales*, leading to the spread of community-acquired and nosocomial infections. The genotypes of ESBL-producing bacteria include TEM, SHV, and CTX-M. In particular, the CTX-M type is spreading worldwide, and a pandemic was declared in 2006 [5,6]. There are more than 160 CTX-M ESBL subtypes that can be classified into subgroups, including the CTX-M-1 group, CTX-M-2 group, and CTX-M-9 group [7]. The distribution of these subtypes varies regionally and temporally. Sequence type 131 (ST131) is the predominant sequence type and is highly pathogenic and multidrug-resistant [8,9]. The presence of CTX-M-15, a common type of ESBL, was responsible for the global outbreak of ESBL-producing *E. coli*, and this type often belongs to ST131. It accounts for 8% of urinary non-ESBL-producing isolates in patients in a single institution in the United States [10]. The epidemic clones of *E. coli* ST131 carry a high number of virulence and resistance genes. Not only resistance to routine antibiotics was reported in *E. coli* ST131, but also resistance to carbapenem was a matter of great concern in this strain. For effective treatment of infections involving ESBL-producing organisms, it is important to properly identify the genotype and use appropriate antimicrobial agents that can preserve carbapenems as an AMR countermeasure [11].

Flomoxef (FMOX) is a broad-spectrum antibiotic with oxygen substituted for sulfur and the 7-α-methoxy group in the cephalosporin core that was introduced to the medical field in the 1980s [12]. The in vitro and clinical efficacy of flomoxef have been proven satisfactory in the treatment of infections caused by both Gram-positive and Gram-negative bacteria with minimal side effects [13,14]. The use of FMOX in empiric and definitive therapy has been previously evaluated and proven to be effective for ESBL-producing *E. coli* [15]. Preclinical studies have reported that FMOX susceptibility among ESBL-producing *E. coli* was comparable to imipenem (IPM) and meropenem (MEPM) [16,17]. Clinical studies conducted in patients with UTIs caused by ESBL-producing *E. coli* showed comparable susceptibility between FMOX and IPM [18,19]. Therefore, FMOX has attracted substantial attention as a promising agent for the treatment of ESBL-producing *E. coli* infections as an alternative to broad-spectrum antibiotics, such as carbapenem. In 2021, the study of the in vitro efficacy of FMOX against ESBL-producing *E. coli* associated with UTIs in Malaysia was reported [20]. In this study, carbapenem remained the most effective antibiotic against ESBL-producing *E. coli* associated with UTIs, followed by FMOX [20]. However, the in vitro activity of FMOX on ESBL-producing *E. coli* with respect to genotypic and phenotypic characteristics has not been investigated. The current literature showed that FMOX would be a potential alternative to broad-spectrum antimicrobial agents for the treatment of UTIs caused by ESBL-producing *E. coli*. Here, we carried out a molecular biological analysis to investigate ESBL-producing *E. coli* strains and the use of FMOX, which has good sensitivity in terms of its minimum inhibitory concentration (MIC).

## 2. Results

### 2.1. E. coli Isolates and Antibiotic Sensitivity

**Figure 1:**
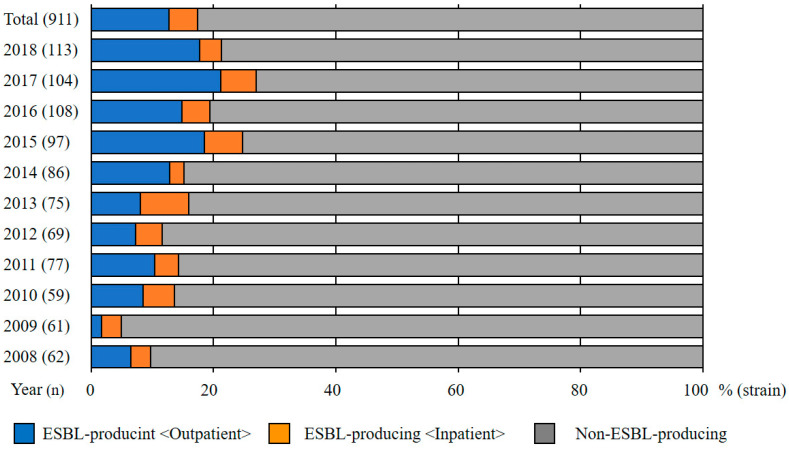
Trends of *E. coli* isolated in urine from urinary tract infections. Among the urine samples collected between 2008 and 2018, *E. coli* strains were isolated from urine, of which 17.3% were ESBL-producing strains. Of these, nearly 73% were from outpatients, and the remainder, 27%, were from inpatients.

Among the urine samples collected between 2008 and 2018, 911 *E. coli* strains were isolated from urine, of which 158 (17.3%) were ESBL-producing (Figure 1). Of these, nearly three-quarters (116/158; 73%) were from outpatients, and the remainder (42/158; 27%) were from inpatients. All 158 ESBL-producing *E. coli* strains were resistant to CEZ, all but one to CFDN, and all but two to CFPM (Table 1). On the other hand, all strains were susceptible to IPM and MEPM, all but one to FOM, all but two to FMOX, all but four to CMZ, and all but ten to PIPC/TAZ. Other strains that showed sensitivity to each drug were 133 (84.2%) for GM, 79 (50%) for CAZ, 66 (41.8%) for ST, and 25 (15.8%) for LVFX.

### 2.2. Multilocus Sequence Typing and β-Lactamase Gene PCR

**Figure 2:**
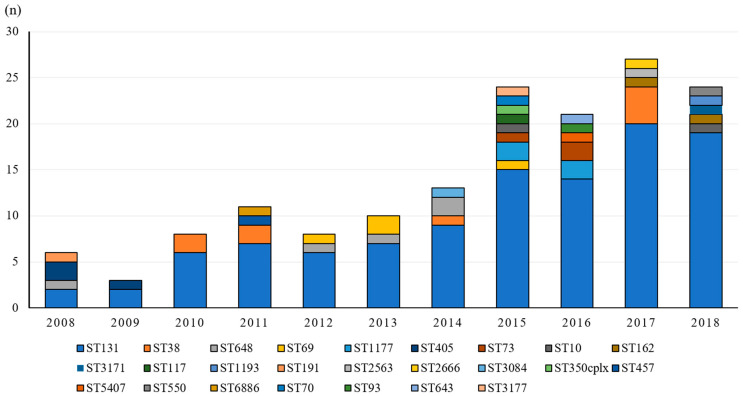
MLST analysis of ESBL-producing *E. coli* strains. ST131 was the most commonly identified strain with 67.7%, and the frequency increased over time. The next most common was ST38, with 5.1%.

Results of MLST analysis of the 158 ESBL-producing *E.coli* strains are shown in Figure 2. ST131 was the most commonly identified strain, with 107 out of 158 (67.7%), and the frequency increased over time. The next most common was ST38 with eight strains (5.1%), and the other strains each had fewer than eight within the panel.

**Figure 3:**
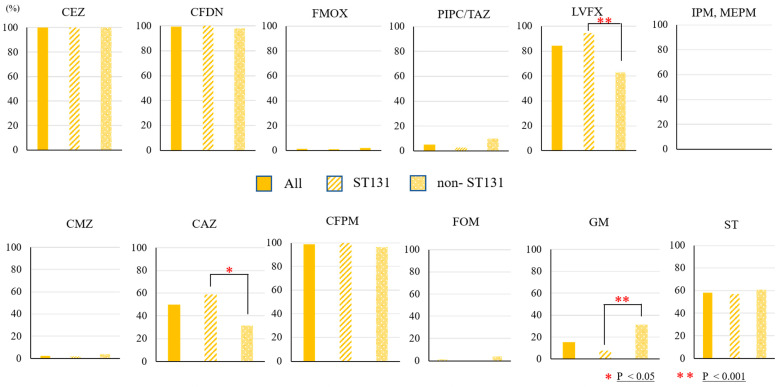
Antimicrobial susceptibility to ST131 and non-ST131 *E. coli*. Among ST131 and non-ST131 ESBL-producing *E. coli*, there were no differences in susceptibility rates to CEZ, CFDN, FMOX, PIPC/TAZ, IPM, MEPM, CMZ, CFPM, FOM, and ST. While there were significant differences in susceptibility rates for LVFX, CAZ, and GM.

Antimicrobial susceptibility to ST131 and non-ST131 *E. coli* was shown (Figure 3). For CEZ, 100% (107/107) of ST131 strains were not susceptible, and 100% (51/51) of non-ST131 strains were not susceptible (*p* = 1.000). For CFDN, 100% (107/107) of ST131 strains were not susceptible, and 98% (50/51) of non-ST131 strains were not susceptible (*p* = 0.323). For FMOX, 93% (1/107) of ST131 strains were not susceptible, and 1.9% (50/51) of non-ST131 strains were not susceptible (*p* = 0.543). For PIPC/TAZ, 2.8% (3/107) of ST131 strains were not susceptible, and 9.8% (5/51) of non-ST131 strains were not susceptible (*p* = 0.113). For LVFX, 94% (101/107) of ST131 strains were not susceptible, and 63% (32/51) of non-ST131 strains were not susceptible (*p* < 0.001). For IPM and MEPM, 0% (0/107) of ST131 strains were not susceptible, and 0% (0/51) of non-ST131 strains were not susceptible (*p* = 1.00). For CMZ, 1.9% (2/107) of ST131 strains were not susceptible, and 3.9% (2/51) of non-ST131 strains were not susceptible (*p* = 0.595). For CAZ, 59% (63/107) of ST131 strains were not susceptible, and 31% (16/51) of non-ST131 strains were not susceptible (*p* = 0.002). For CFPM, 100% (107/107) of ST131 strains were not susceptible, and 96% (49/51) of non-ST131 strains were not susceptible (*p* = 0.103). For FOM, 0% (0/107) of ST131 strains were not susceptible, and 3.9% (2/51) of non-ST131 strains were not susceptible (*p* = 0.103). For GM, 7.5% (8/107) of ST131 strains were not susceptible, and 31% (16/51) of non-ST131 strains were not susceptible (*p* < 0.001). For ST, 57% (61/107) of ST131 strains were not susceptible, and 61% (31/51) of non-ST131 strains were not susceptible (*p* = 0.731).

**Figure 4:**
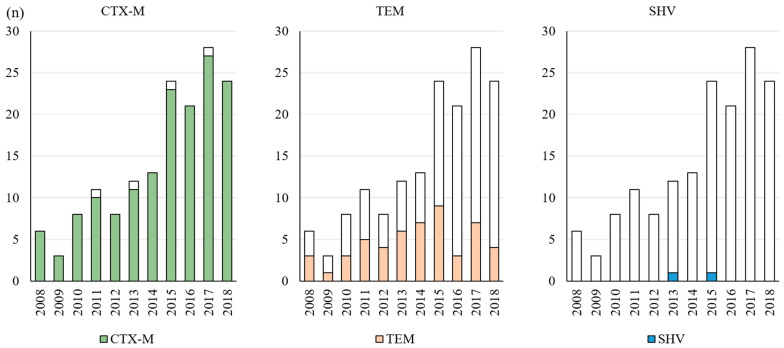
Genotype. In genotyping using multiplex PCR, the CTX-M type was the most common genotype, accounting for 97.5%, followed by the TEM type with 32.9%. However, only 1.3% were SHV types.

In genotyping using multiplex PCR (Figure 4), the CTX-M type was the most common genotype, accounting for 154 of 158 isolates (97.5%), followed by the TEM type with 52 of 158 (32.9%). However, only 2 of 158 isolates (1.3%) were SHV-type.

**Figure 5:**
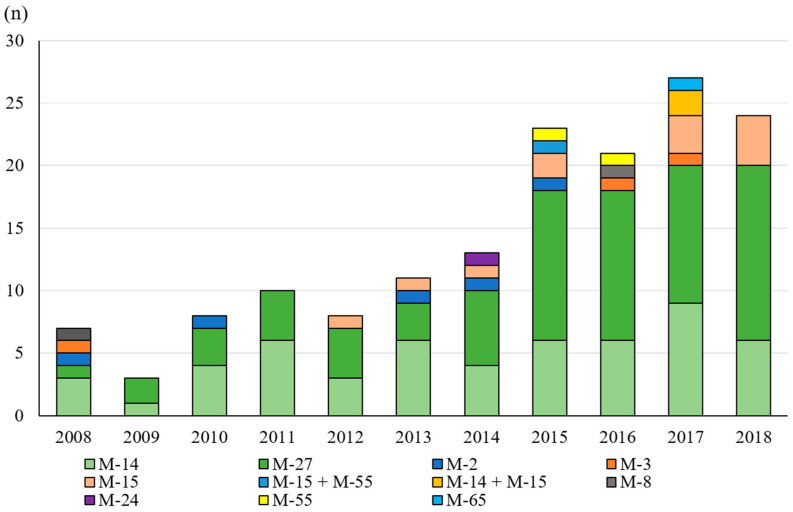
The classification of the CTM-M type. For the classification of the CTX-M type, the CTX-M-9 group members, CTX-M-14 type, and the CTX-M-27 type predominated among the study samples. CTX-M-14 type was predominant in 35.1% of strains, and CTX-M-27 type was seen in 46.8% of strains. The CTX-M-14 type predominated through 2013, and the CTX-M-27 type came to dominate in 2014. The next most common type was CTX-M-15, which belongs to the CTX-M-1 group, with 7.8%.

The classification of the CTX-M type is shown in Figure 5. For the classification of the CTX-M type, the CTX-M-9 group members CTX-M-14 type and CTX-M-27 type predominated among the study samples. The CTX-M-14 type was predominant in 54 of 154 (35.1%) strains, and the CTX-M-27 type was seen in 72 of 154 (46.8%) strains. The CTX-M-14 type predominated through 2013, while the CTX-M-27 type predominated in 2014. The next most common type was CTX-M-15, which belongs to the CTX-M-1 group, with 12 of 154 (7.8%).

**Figure 6:**
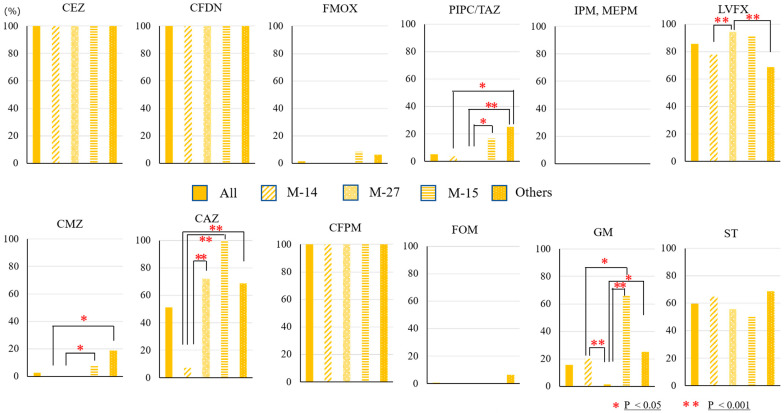
Antimicrobial susceptibility to *E. coli* with the CTX-M type. Among the CTX-M types, no differences in susceptibility to CEZ, CFDN, FMOX, IPM, MEPM, CFPM, FOM, and ST were observed. However, significant differences in susceptibility to PIPC/TAZ, LVFX, CMZ, CAZ, and GM were observed.

Antimicrobial susceptibility to CTX-M types in *E. coli* was shown (Figure 6). For CEZ, 100% (54/54) of CTX-M-14 strains were not susceptible, 100% (72/72) of CTX-M-27 strains were not susceptible, 100% (12/12) of CTX-M-15 strains were not susceptible, and 100% (16/16) of other strains were not susceptible. For CFDN, 100% (54/54) of CTX-M-14 strains were not susceptible, 100% (72/72) of CTX-M-27 strains were not susceptible, 100% (12/12) of CTX-M-15 strains were not susceptible, and 100% (16/16) of other strains were not susceptible. For FMOX, 0% (0/54) of CTX-M-14 strains were not susceptible, 0% (0/72) of CTX-M-27 strains were not susceptible, 8.3% (1/12) of CTX-M-15 strains were not susceptible, and 100% (6.3/16) of other strains were not susceptible. For PIPC/TAZ, 3.7% (2/54) of CTX-M-14 strains were not susceptible, 0% (0/72) of CTX-M-27 strains were not susceptible, 17% (2/12) of CTX-M-15 strains were not susceptible, and 25% (4/16) of other strains were not susceptible. For IPM and MEPM, 3.7% (2/54) of CTX-M-14 strains were not susceptible, 0% (0/72) of CTX-M-27 strains were not susceptible, 17% (2/12) of CTX-M-15 strains were not susceptible, and 25% (4/16) of other strains were not susceptible. For LVFX, 78% (42/54) of CTX-M-14 strains were not susceptible, 94% (68/72) of CTX-M-27 strains were not susceptible, 92% (11/12) of CTX-M-15 strains were not susceptible, and 69% (11/16) of other strains were not susceptible. For CMZ, 0% (0/54) of CTX-M-14 strains were not susceptible, 0% (0/72) of CTX-M-27 strains were not susceptible, 8.3% (1/12) of CTX-M-15 strains were not susceptible, and 19% (3/16) of other strains were not susceptible. For CAZ, 7.4% (4/54) of CTX-M-14 strains were not susceptible, 72% (52/72) of CTX-M-27 strains were not susceptible, 100% (12/12) of CTX-M-15 strains were not susceptible, and 69% (11/16) of other strains were not susceptible. For CFPM, 100% (54/54) of CTX-M-14 strains were not susceptible, 100% (72/72) of CTX-M-27 strains were not susceptible, 100% (12/12) of CTX-M-15 strains were not susceptible, and 100% (16/16) of other strains were not susceptible. For FOM, 0% (0/54) of CTX-M-14 strains were not susceptible, 0% (0/72) of CTX-M-27 strains were not susceptible, 0% (0/12) of CTX-M-15 strains were not susceptible, and 6.3% (1/16) of other strains were not susceptible. For GM, 20% (11/54) of CTX-M-14 strains were not susceptible, 1.4% (1/72) of CTX-M-27 strains were not susceptible, 67% (8/12) of CTX-M-15 strains were not susceptible, and 25% (4/16) of other strains were not susceptible. For ST, 65% (35/54) of CTX-M-14 strains were not susceptible, 56% (40/72) of CTX-M-27 strains were not susceptible, 50% (6/12) of CTX-M-15 strains were not susceptible, and 69% (11/16) of other strains were not susceptible.

Two ESBL-producing *E. coli* strains that were resistant to FMOX are described in Table 2. The first strain had a ST131 sequence type and genotype of CTX-M-2 and was susceptible to PIPC/TAZ, IPM, MEPM, FOM, and GM. The other strain was a ST354 sequence type, had CTX-M-15 and CMY161 genotypes, and was susceptible to IPM, MEPM, and FOM. As mentioned above, the two FMOX-resistant strains had no correlation between ST analysis and genotype. As can be seen from the MIC, both strains were also resistant to CMZ. Furthermore, FMOX had greater efficacy than CMZ based on MIC_50_ and MIC_90_ values (Table 1).

### 2.3. The Clinical Efficacy of FMOX

**Figure 7:**
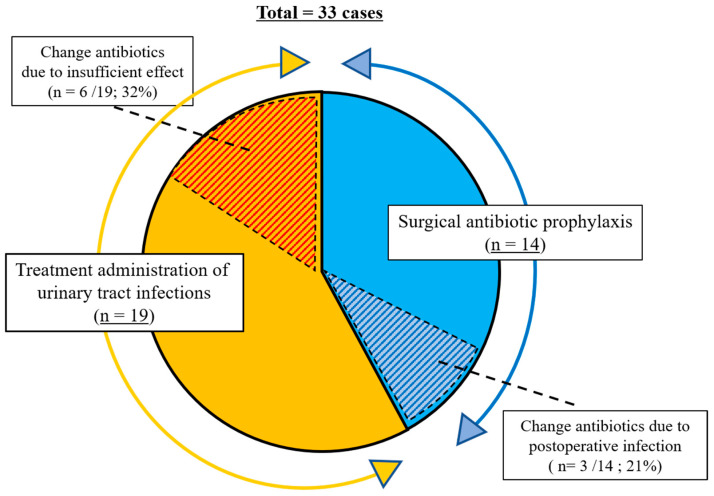
The breakdown of cases treated with FMOX. Fourteen were for prophylactic administration prior to surgery, and 19 were for therapeutic administration for UTI. Of the 14 cases with prophylactic administration, 3 were switched to other agents due to postoperative infection, and of the 19 cases that received therapeutic administration, 6 were switched due to inadequate efficacy.

The breakdown of cases treated with FMOX in our study is shown in Figure 7. Among the 33 cases that received FMOX at our institution (Figure 7), 14 were for prophylactic administration prior to surgery and 19 were for therapeutic administration for UTI. Of the 14 cases with prophylactic administration, 3 were switched to other agents due to postoperative infection, and of the 19 cases that received therapeutic administration, 6 were switched due to inadequate efficacy.

Of the 33 cases treated with FMOX at our institution (Table 3), 16 had *E. coli*, three had *Proteus mirabilis*, and one each had *Klebsiella oxytoca*, *Citrobacter koseri*, *Pseudomonas aeruginosa*, and *MRSA*. Two had other Gram-negative rods, two had *Enterococcus* spp, one had Gram-positive cocci, and three were culture-negative. There were a total of eight ESBL-producing bacteria identified: five *E. coli* and one each of *Klebsiella oxytoca*, *Proteus mirabilis*, and *Citrobacter koseri* (Table 4). For the five cases with *E. coli*, three received FMOX for surgical prophylaxis against urinary ESBL-producing *E. coli*, and all treatments successfully prevented postoperative infections. On the other hand, two patients received FMOX to treat UTIs: one patient had an effective response, and the other had an inadequate response and was switched to carbapenems.

## 3. Materials and Methods

### 3.1. ESBL-Producing E. coli

*E. coli* isolated from the urine of patients with UTI who were treated at the outpatient clinic or in a ward of Okayama University Hospital, Okayama, Japan, between January 2008 and December 2018 were included in our study. Pyuria was diagnosed as the presence of white blood cells (WBC) ≥5/HPF in a urine sediment specimen, and bacteriuria was diagnosed as a bacterial count ≥10^3^ CFU/mL in catheter urine or 10^4^ CFU/mL in midstream urine. Isolates were counted as one infection episode per patient, and plural isolates were counted in polymicrobial infections.

ESBL production of *E. coli* was confirmed using the disc method carried out according to Clinical and Laboratory Standards Institute (CLSI) document M100-S22 [21] with 30 μg/mL CTX. A total of 30 ESBL-producing *E. coli* strains were identified when the inhibition circle had a diameter larger than 5 mm using 30 μg/mL CTX, 30 μg/mL CTX plus 10 μg/mL CVA, 30 μg/mL CAZ, or 30 μg/mL CAZ plus 10 μg/mL CVA.

Sequence type analysis by multilocus sequence typing (MLST) and classification of ESBL genotypes by Multiplex PCR were performed on ESBL-producing *E. coli* strains. MLST is a molecular genetic method that involves the sequencing of seven housekeeping genes (adk, fumC, gyrB, icd, mdh, purA, and recA) to determine allele numbers that are then compared with information in enterobase (https://enterobase.warwick.ac.uk accessed on 1 March 2018). The sequencing type is determined by combining the seven types of genes (Table 5).

Classification of ESBL genotypes by Multiplex PCR was performed according to the used primers as described by Dallenne et al. [22]. Rapid DNA preparation was carried out by first placing one colony in 100 µL distilled water and heating at 95 °C. Total DNA (2 µL) was subjected to multiplex PCR in a 50 µL reaction mixture containing 1× PCR buffer (10 mM Tris-HCl, pH 8.3/50 mM KCl/1.5 mM MgCl_2_), 200 mM of each deoxynucleotide triphosphate, a variable concentration of group-specific primers (Table 6), and 1 U *Taq* polymerase (Sigma Aldrich, St Quentin Fallavier, France). DNA was amplified with initial denaturation at 94 °C for 10 min followed by 30 cycles of 94 °C for 40 s, 60 °C for 40 s and 72 °C for 1 min, and a final 7-min elongation step at 72 °C. A 55 °C annealing temperature was used to amplify the bla_VIM_, bla_IMP_, and bla_KPC_ genes, and 57 °C was used to amplify the bla_GES_ and bla_OXA-48_ genes. Amplicons were visualized on a 2% agarose gel containing ethidium bromide run at 100 V for 1 h. A 100-bp DNA ladder (New England Biolabs, Ipswich, MA, USA) was used as a size marker. To identify the β-lactamase genes detected in the multiplex PCR assays, DNA sequence analyses of the amplicons were performed. Amplified PCR products were purified using the ExoSap purification kit (ExoSap-it, GE Healthcare, Piscataway, NJ, USA), and bidirectional sequencing was performed. Each sequence is aligned by multiple-sequence alignment using the BLAST program.

The minimum inhibitory concentrations (MIC) of various antimicrobials were analyzed using micro-liquid dilution and agar dilution methods, and drug susceptibility was measured. Antimicrobial agents for which MICs were measured were cefazolin (CEZ), cefdinir (CFDN), flomoxef (FMOX), cefmetazole (CMZ), ceftazidime (CAZ), cefepime (CFPM), piperacillin/tazobactam (PIPC/TAZ), imipenem (IPM), faropenem (FRPM), meropenem (MEPM), levofloxacin (LVFX), sitafloxacin (STFX), fosfomycin (FOM), gentamicin (GM), sulfamethoxazole/trimethoprim (ST).

### 3.2. Flomoxef Efficacy

To investigate the efficacy of flomoxef (FMOX) against ESBL-producing *E. coli*, samples from 33 patients with UTI who were treated with FMOX at our institution between 2008 and 2018 were collected. The rationale for the choice of FMOX, urinary isolates, drug sensitivity, administration method, and outcome were retrospectively reviewed from patient charts to determine the clinical positioning of FMOX.

### 3.3. Statistical Analyses

Statistical analyses were performed using EZR software (version 1.71; Saitama Medical Center, Jichi Medical University, Saitama, Japan) [23]. The antimicrobial susceptibilities among the strains were compared with Fisher’s exact test. Results with *p* values < 0.05 were considered statistically significant.

## 4. Discussion

In our study, the susceptibility of ESBL-producing *E. coli* to FMOX was comparable to that seen for MEPM and TAZ/PIPC. Among the *E. coli* strains, ST131 was the most common sequence type (67.7%), and in our study population, the percentage increased annually. Consistent with previous reports, CTX-M was the most common genotype, and the frequency of CTX-M-27 increased over that of CTX-M-14 after 2013; CTX-M-15 appeared in 2012. The susceptibility rate of CTX-M-15 to FMOX, TAZ/PIPC, CMZ, CAZ, and GM was lower than that for other genotypes.

ESBL-producing *E. coli* were first reported by Knothe et al. in 1983 [24], and ESBL-producing *E. coli* were isolated in Japan by Ishii et al. in 1993 [25]. TEM and SHV types of ESBL-producing *E. coli* were prevalent in Europe and the United States in the 1980s, and CTX-M types spread worldwide beginning in 2000; a pandemic of these types was declared in 2006 [5]. Since 2010, CTX-M-27 and CTX-M-15 have been detected in Japan and abroad [26,27]. These phenotypes tend to be more resistant to drugs than CTX-M-14, which was the mainstay of AMR [28]. In our study, we also observed increased resistance rates to LVFX, CAZ, and GM by CTX-M-27 and CTX-M-15 compared to CTX-M-14, which is comparable to earlier findings. In addition, the frequencies of TEM and SHV were similar to those reported by Aihe et al. [29], but for TEM, the frequency was lower than that seen by Jena et al. [30]. There has been almost no genotype analysis of UTIs in Japan, and such studies would be useful for better understanding the prevalence of multidrug-resistant *E. coli* in the country. Moreover, since 2000, ST131 has become the predominant *E. coli* isolate worldwide [8], and our results were consistent with this trend. The ST131 group had a higher rate of insensitivity to LVFX and CAZ compared to non-ST131 strains (Figure 3). Among antimicrobial agents, fluoroquinolone resistance is increasing [31], and of the 158 isolates in this study, the majority (133/158; 84.2%) were resistant to LVFX. The most promising antimicrobial agents are carbapenems [32], and other antimicrobial agents, including TAZ/PIPC, CMZ, and FMOX, continue to have good activity [33,34]. FOM is reported to be effective in the US and Europe [35], and the new drug TAZ/CTLZ is also expected to be effective [36]. We observed a similar trend in our study. However, due to the risk of AMR, the use of carbapenems and TAZ/PIPC should be limited whenever possible, and CMZ and FMOX should be used with preference.

We also examined the clinical status of FMOX, and our findings suggest that FMOX has some efficacy for perioperative prophylactic administration in urologic surgery for carriers of ESBL-producing bacteria and for therapeutic administration for UTIs. FMOX is an oxacefem-derived antimicrobial agent that was first released in 1988 and is effective against Gram-negative rods, MSSA, and anaerobes, including *E. coli*. FMOX has also been reported to be effective against ESBL-producing bacteria [24] and ESBL-producing *E. coli* [34]. In addition, FMOX has efficacy against CTX-M-type ESBL-producing bacteria with urinary excretion [16,37] and for UTIs caused by ESBL-producing *E. coli* in children [18]. The MIC data for both IPN and MEPM look interesting and are as good as those for FMOX in the present study. In fact, in a clinical situation, a previous study showed that no significant differences were observed in the febrile period and recurrence rate between the FMOX-initiated group and the other antibiotic groups, suggesting that FMOX may be a useful alternative carbapenem antibiotic for UTIs caused by ESBL-producing *E. coli* [38]. The cause of the change in the percentage of ESBLs within the observation period is unknown, but the effect of changes in therapeutic agents is less likely, and no severe cases were observed in the current reports. We think that FMOX would be a suitable alternative for perioperative prophylactic administration because of its narrow-spectrum antimicrobial feature, therapeutic administration for UTIs caused by ESBL-producing bacteria of moderate or lower severity, and de-escalation from empiric therapy against ESBL-producing bacteria. This approach would allow for more limited use of carbapenem and combination β-lactamase inhibitors as part of AMR countermeasures.

Uropathogenic *E. coli* strains have a variety of flexible and adaptive genomic pools. These genomic pools are the arsenal of a wide range of virulence and drug resistance genes. Therefore, the armed uropathogenic *E. coli* pathotypes with a powerful virulome enable them to survive against the human body’s immune system and the deathly effects of antimicrobial agents. However, there is neither a regular pattern for drug resistance properties nor ESBL production in reported isolated pathotypes of Uropathogenic *E. coli* worldwide. For example, the presence or absence of *mrk* genes has no correlation with antimicrobial susceptibility in uropathogenic *E. coli* pathotypes [39]. On the other hand, the presence of *hlyA* and *cnf1* virulence genes may lead to increased bacterial sensitivity to fluoroquinolones [40]. The gene profiles may be helpful for choosing the appropriate antibiotic for determined uropathogenic *E. coli* strains in the procedure for the treatment of UTIs. Further study is needed to determine the sensitivity of FOMX from the perspective of gene profiles.

The present study had some limitations. First, this study was done in a single hospital in Japan, and due to budget limitations, a multicenter study was not possible. Access to medical data of the patients, such as underlying patients characteristics, diseases, and recent medications, to correlate their links with colonization of *E. coli* strains and their antimicrobial resistance phenotypes was not possible, because of the lack of registry system. In addition, there was no follow-up program at the time of the study to understand differences in the success or complications of UTIs caused by *E. coli* identified in our hospital. Although our strain pools could not represent the entire ESBL-producing *E. coli* population in this region, both temporally and geographically, the data generated in the present study provided an insight into the extent of β-lactam resistance among ESBL producers associated with UTIs in Japan. Moreover, the susceptibility data obtained in this study support further consideration of the potential use of FMOX as an alternative option for the treatment of UTIs caused by ESBL-producing *E. coli*. Second, the major drawback of the present study was that the analyzed isolates were collected from 2008 to 2018 and did not reflect the current situation well. However, it is meaningful work to provide the basic molecular characteristics of a common causative pathogen for community-based UTIs. Further studies on changes in microbiological characteristics in Japan are necessary to clarify this issue.

Despite these limitations, our results showed ESBL-producing *E. coli* isolates with common rep-types presented diversity in their clone types and antibiotic-resistance patterns. Constant monitoring would be done to control their spread in the community because of the high prevalence of the strains and their involvement in UTIs.

## 5. Conclusions

The frequency of ESBL-producing *E. coli* is increasing annually, especially in outpatients. Although antimicrobial susceptibility has not significantly decreased, we have continued to observe *E. coli* strains like CTX-M-15 that have a multidrug-resistant genotype. Throughout the study period, favorable sensitivity to FMOX was observed, suggesting that FMOX continues to be a valid treatment option that can prevent AMR. We think that the continuous surveillance of ESBL-producing *E. coli* at both the institutional and national levels will preserve the clinical efficacy of broad-spectrum cephalosporin and prevent the emergence of carbapenem resistance in local *E. coli* strains.

## Figures and Tables

**Table 1:** Antibiotic susceptibility to different antibiotic agents.

Antibiotic Agent	MIC (μg/ml) for All Isolates (n = 158)			Susceptible Rate (%)			
	Breakpoint ^*^	MIC_50_	MIC_90_	2008–2010	2011–2013	2014–2016	2017–2018
				(n = 17)	(n = 31)	(n = 58)	(n = 52)
cefazolin	2	>16	>16	0	0	0	0
cefdinir	1	>8	>8	0	0	2	0
flomoxef	8 ^†^	0.125	0.5	94	100	100	98
cefmetazole	16	2	8	88	100	98	98
ceftazidime	4	4	32	59	65	45	44
cefepime	2	>8	>8	0	3	2	0
piperacilin/tazobactam	16/4	2/4	16/4	83	97	97	92
imipenem	1	≦0.5	≦0.5	100	100	100	100
faropenem	- ^‡^	0.5	2	-	-	-	-
meropenem	1	≦0.5	≦0.5	100	100	100	100
levofloxacin	0.5	16	32	12	16	22	10
sitafloxacin	-^‡^	1	2	-	-	-	-
fosfomycin	64	4	8	100	100	97	100
gentamicin	4	≦1	>16	88	81	84	85
sulfamethoxazole/trimethoprim	2/38	8	>64	24	42	50	38

^*^: CLSI M100-ED30:based on 2020 breakpoint; ^†^: Breakpoint of flomoxef is 8μg/ml (J Infect Chemother. 2017 Aug;23(8):517-522.) [18]; ^‡^: fBreakpoint is not defined about faropenem, sitafloxacin.

**Table 2:** Two ESBL-producing *Escherichia coli* strains that are resistant to FMOX.

No.	ST	β-Lactamase Gene PCR	MIC_50 (µg/mL)_		Antibiotic Agent Sensitivity
			FMOX	CMZ	
1	ST131	CTX-M-2	16	64	PIPC/TAZ, IPM, MEPM, FOM, GM
2	ST354	CTX-M-15,CMY161	64	>64	IPM, MEPM, FOM

**Table 3:** Bacteria types were identified in urine samples collected before administration of FMOX.

Bacteria Types	*n*
*Escherichis* *coli*	16
ESBL-producing	5
not ESBL-producing	11
*Klebsiella oxytoca* (ESBL-producing)	1
*Proteus mirabillis*	3
ESBL-producing	1
not ESBL-producing	2
*Citrobacter koseri* (ESBL-producing)	1
*Pseudomonas aeruginosa*	1
Other Gram-negative rods	2
Enterococcus	2
MRSA	1
Other Gram-positive cocci	3
Negative	3

**Table 4:** Outcomes for treatment with FMOX against ESBL-producing bacteria.

	Clinically Effective (Success)	Change to Other Antibiotic (Failure)	Other Antibiotic
Surgical antibiotic prophylaxis			
*Escherichia coli* ESBL-producing	3	-	
Other ESBL-producing bacteria	-	1	DRPM
Urinary tract infection treatment			
*Escherichia coli* ESBL-producing	1	1	DRPM, STFX
Other ESBL-producing bacteria	1	1	DRPM

**Table 5:** Primers and reaction conditions used for MLST.

Primer	Target Gene	Nucleotide Sequence (5′---3′)	Denaturation	Annealing	Expansion	Number of Cycles	Product Length (b.p.)
adkF	adk	ATTCTGCTTGGCGCTCCGGG	95 °C 1 min	54 °C 1 min	72 °C 2 min	30	583
adkR		CCGTCAACTTTCGCGTATTT					
fumCF	fumC	TCACAGGTCGCCAGCGCTTC		54 °C 1 min			806
fumCR		GTACGCAGCGAAAAAGATTC					
gyrBF	gyrB	TCGGCGACACGGATGACGGC		60 °C 1 min			911
gyrBR		ATCAGGCCTTCACGCGCATC					
icdF	icd	ATGGAAAGTAAAGTAGTTGTTCCGGCACA		54 °C 1 min			878
icdR		GGACGCAGCAGGATCTGTT					
mdhF	mdh	ATGAAAGTCGCAGTCCTCGGCGCTGCTGGCGG		60 °C 1 min			932
mdhR		TTAACGAACTCCTGCCCCAGAGCGATATCTTTCTT					
purAF	purA	CGCGCTGATGAAAGAGATGA		54 °C 1 min			816
purAR		CATACGGTAAGCCACGCAGA					
recAF	recA	CGCATTCGCTTTACCCTGACC		58 °C 1 min			780
recAR		TCGTCGAAATCTACGGACCGGA					

**Table 6:** Group-specific primers used for the assays.

PCR Name	β-Lactamase Targeted	Primer Name	Sequence(5′-3′)	Length (Bases)	Annealing Position	Amplicon Size(bp)	Primer Concentration
							(pmol/μL)
Multiplex I TEM, SHV and OXA-1-like	TEM variants including TEM-1 and TEM-2	MultiTSO-T_for	CATTTCCGTGTCGCCCTTATTC	22	13–34	800	0.4
		MultiTSO-T_rev	CGTTCATCCATAGTTGCCTGAC	22	812–791		0.4
	SHV variants including SHV-1	MultiTSO-S_for	AGCCGCTTGAGCAAATTAAAC	21	71–91	713	0.4
		MultiTSO-S_rev	ATCCCGCAGATAAATCACCAC	21	783–763		0.4
	OXA-1, OXA-4 and OXA-30	MultiTSO-O_for	GGCACCAGATTCAACTTTCAAG	22	201–222	564	0.4
		MultiTSO-O_rev	GACCCCAAGTTTCCTGTAAGTG	22	764–743		0.4
Multiplex II CTX-M group 1, group 2 and group 9	variants of CTX-M group 1 including CTX-M-1, CTX-M-3 and CTX-M-15	MultiCTXMGp1_for	TTAGGAARTGTGCCGCTGYA ^b^	20	61–80	688	0.4
		MultiCTXMGp1-2_rev	CGATATCGTTGGTGGTRCCAT ^b^	21	748–728		0.2
	variants of CTX-M group 2 including CTX-M-2	MultiCTXMGp2_for	CGTTAACGGCACGATGAC	18	345–362	404	0.2
		MultiCTXMGp1-2_rev	CGATATCGTTGGTGGTRCCAT ^b^	21	748–728		0.2
	variants of CTX-M group 9 including CTX-M-9 and CTX-M-14	MultiCTXMGp9_for	TCAAGCCTGCCGATCTGGT	19	299-317	561	0.4
		MultiCTXMGp9_rev	TGATTCTCGCCGCTGAAG	18	859–842		0.4
CTX-M group 8/25	CTX-M-8, CTX-M-25, CTX-M-26 and CTX-M-39 to CTX-M-41	CTX-Mg8/25_for	AACRCRCAGACGCTCTAC ^b^	18	172–189	326	0.4
		CTX-Mg8/25_rev	TCGAGCCGGAASGTGTYAT ^b^	19	497–479		0.4
Multiplex III ACC, FOX, MOX, DHA, CIT and EBC	ACC-1 and ACC-2	MultiCaseACC_for	CACCTCCAGCGACTTGTTAC	20	744–763	346	0.2
		MultiCaseACC_rev	GTTAGCCAGCATCACGATCC	20	1089–1070		0.2
	FOX-1 to FOX-5	MultiCaseFOX_for	CTACAGTGCGGGTGGTTT	18	396–413	162	0.5
		MultiCaseFOX_rev	CTATTTGCGGCCAGGTGA	18	557–540		0.5
	MOX-1, MOX-2, CMY-1, CMY-8 to CMY-11 and CMY-19	MultiCaseMOX_for	GCAACAACGACAATCCATCCT	21	3–23	895	0.2
		MultiCaseMOX_rev	GGGATAGGCGTAACTCTCCCAA	22	900–879		0.2
	DHA-1 and DHA-2	MultiCaseDHA_for	TGATGGCACAGCAGGATATTC	21	113–133	997	0.5
		MultiCaseDHA_rev	GCTTTGACTCTTTCGGTATTCG	22	1109–1088		0.5
	LAT-1 to LAT-3, BIL-1, CMY-2 to CMY-7, CMY-12 to CMY-18 and CMY-21 to CMY-23	MultiCaseCIT_for	CGAAGAGGCAATGACCAGAC	20	570–589	538	0.2
		MultiCaseCIT_rev	ACGGACAGGGTTAGGATAGY ^b^	20	1107–1088		0.2
	ACT-1 and MIR-1	MultiCaseEBC_for	CGGTAAAGCCGATGTTGCG	19	189–207	683	0.2
		MultiCaseEBC_rev	AGCCTAACCCCTGATACA	18	871–854		0.2
Multiplex IV VEB, PER and GES	GES-1 to GES-9 and GES-11	MultiGES_for	AGTCGGCTAGACCGGAAAG	19	463–481	399	0.3
		MultiGES_rev	TTTGTCCGTGCTCAGGAT	18	861–844		0.3
	PER-1 and PER-3	MultiPER_for	GCTCCGATAATGAAAGCGT	19	325–343	520	0.3
		MultiPER_rev	TTCGGCTTGACTCGGCTGA	19	844–826		0.3
	VEB-1 to VEB-6	MultiVEB_for	CATTTCCCGATGCAAAGCGT	20	187–206	648	0.3
		MultiVEB_rev	CGAAGTTTCTTTGGACTCTG	20	834–815		0.3
Multiplex V GES and OXA-48-like	GES-1 to GES-9 and GES-11	MultiGES_for	AGTCGGCTAGACCGGAAAG	19	463–481	399	0.3
		MultiGES_rev	TTTGTCCGTGCTCAGGAT	18	861–844		0.3
	OXA-48-like	MultiOXA-48_for	GCTTGATCGCCCTCGATT	18	230–247	281	0.4
		MultiOXA-48_rev	GATTTGCTCCGTGGCCGAAA	20	490–510		0.4
Multiplex VI IMP, VIM and KPC	IMP variants except IMP-9, IMP-16, IMP-18, IMP-22 and IMP-25	MultiIMP_for	TTGACACTCCATTTACDG ^b^	18	194–211	139	0.5
		MultiIMP_rev	GATYGAGAATTAAGCCACYCT ^b^	21	332–313		0.5
	VIM variants including VIM-1 and VIM-2	MultiVIM_for ^c^	GATGGTGTTTGGTCGCATA	19	151–169	390	0.5
		MultiVIM_rev ^c^	CGAATGCGCAGCACCAG	17	540–524		0.5
	KPC-1 to KPC-5	MultiKPC_for	CATTCAAGGGCTTTCTTGCTGC	22	209–230	538	0.2
		MultiKPC_rev	ACGACGGCATAGTCATTTGC	20	746–727		0.2

## Data Availability

Data sharing is not applicable to this study as no datasets were generated.
